# Standardized Hepatitis B Virus RNA Quantification in Untreated and Treated Chronic Patients: a Promising Marker of Infection Follow-Up

**DOI:** 10.1128/spectrum.02149-21

**Published:** 2022-04-04

**Authors:** Maria Francesca Cortese, Mar Riveiro-Barciela, David Tabernero, Francisco Rodriguez-Algarra, Adriana Palom, Sara Sopena, Ariadna Rando-Segura, Luisa Roade, Alison Kuchta, Roser Ferrer-Costa, Josep Quer, Beatriz Pacin, Marta Vila, Rosario Casillas, Selene Garcia-Garcia, Rafael Esteban, Tomás Pumarola, Maria Buti, Francisco Rodriguez-Frias

**Affiliations:** a Clinical Biochemestry, Vall D'hebron Research Institute, Universitat Autònoma de Barcelona, Barcelona, Spain; b Biochemistry and Microbiology, Liver Pathology Unit, Vall D'hebron University Hospital, Universitat Autònoma de Barcelona, Barcelona, Spain; c Centro De Investigación Biomédica En Red, Enfermedades Hepáticas y Digestvas (CIBERehd), Instituto De Salud Carlos III, Madrid, Spain; d Liver Unit, Internal Medicine Department, Vall D'hebron University Hospital, Universitat Autònoma de Barcelona, Barcelona, Spain; e The Blizard Institute, Barts and the London School of Medicine and Dentistry, Queen Mary University of London, London, United Kingdom; f Virology Unit, Microbiology Department, Vall D'hebron University Hospital, Universitat Autònoma de Barcelona, Barcelona, Spain; g Roche Molecular Systems, Inc., Pleasanton, California, USA; h Department of Biochemistry, Vall D'Hebron University Hospital, Barcelona, Spain; i Digestive and Liver Disease, Vall D'hebron Research Institute, Universitat Autònoma de Barcelona, Barcelona, Spain; l Universitat Autònoma de Barcelona, Bellaterra, Spain; Johns Hopkins Hospital

**Keywords:** HBV, HBV-RNA, RT-PCR, antiviral treatment, biomonitoring

## Abstract

The measurement and interpretation of HBV DNA and RNA levels in HBV infected patients treated with antiviral therapy supports the objective of HBV disease management. Here, we quantified circulating HBV RNA through a standardized and sensitive assay in follow-up samples from both naive and treated patients as a marker of infection evolution. HBV DNA (HBV DNA for use in Cobas 6800/8800 Automated Roche Molecular Systems), RNA (Roche HBV RNA Investigational Assay for use in the Cobas 6800/8800; Roche), HBeAg and HBsAg (Elycsys HBsAg chemiluminescence immunoassay by Cobas 8000; Roche), and core-related antigen (Lumipulse G chemiluminescence assay; Fujirebio) levels were measured in cohorts of untreated or nucleos(t)ide treated, HBV-infected subjects in an outpatient hospital setting. HBV DNA levels in untreated people were 3.6 log_10_ higher than corresponding RNA levels and were stable over 5 years of observation. While only five of 52 treated patients had DNA levels below the lower limit of quantification (10 IU/mL) at the end of follow-up, 13 had HBV RNA levels persistently above this limit, including eight with undetectable DNA. In samples with undetectable core-related antigen we observed a median HBsAg titer 2.7-fold higher than in samples with undetectable RNA (adjusted *P* = 0.012). Detectable HBV RNA with undetectable HBV DNA was a negative predictor of HBsAg decrease to a level ≤100 IU/mL (*P* = 0.03). In naive patients the difference between HBV DNA and RNA was higher than previously reported. HBV RNA rapidly decreased during treatment. However, in some cases, it was detectable even after years of effective therapy, being a negative predictor of HBsAg decrease. The investigational RNA assay for use on the Cobas 6800/8800 instruments is a sensitive and standardized method that could be applied in general management of HBV infection.

**IMPORTANCE** This study focused on the quantification of circulating HBV RNA by using a standardized and sensitive assay. Thanks to this system we observed a higher difference between circulating HBV DNA and RNA than previously reported. In treated patients, HBV RNA decreased together with DNA, although some patients presented detectable levels even after years of successful antiviral treatment, suggesting a persistent viral transcription. Of note, the detection of viral RNA when HBV DNA is undetectable was a negative predictor of HBsAg decrease to a level ≤100 IU/mL. This assay could be extremely helpful in HBV patients management to study viral transcription and to identify those treated patients that may achieve sustained viral suppression.

## INTRODUCTION

Over 250 million people worldwide are living with chronic hepatitis B virus infection (HBV) (1), which is the leading cause of hepatocellular carcinoma (HCC) worldwide. It has been estimated that by 2030, 17 million people could die due to chronic HBV-related complications (2).

The HBV life cycle involves the generation of a molecular intermediate, covalently closed circular DNA (cccDNA) that persists in the nucleus of infected hepatocytes. This molecule acts as a template for the synthesis of pregenomic RNA (pgRNA), which is reverse transcribed in nascent viral capsids to synthesize new viral DNA genomes. The establishment of the cccDNA molecules in hepatocyte nuclei generates a persistent infection. Viral DNA can also be randomly integrated into the cellular genome. Due to its linearization and the activity of cellular DNA repair at integration, inserted HBV DNA is truncated (3) and, together with episomal DNA, are the source of continued production of surface antigen (HBsAg) (4), which assembles into subviral particles that are up to 10,000 times more abundant than infectious virions (5, 6).

Treatment of HBV-infected patients with nucleos(t)ide analog (NA) antivirals results in suppression of viral replication and long-term suppression of HBV DNA in most patients. Loss of HBsAg in the blood (functional cure) is the primary goal of antiviral therapy (7) but is achieved in a very limited number of patients because available NAs do not interfere with transcription of pgRNA from integrated DNA or cccDNA, despite suppression of HBV DNA replication. Because integrated HBV DNA also produces HBsAg, HBsAg might not be an adequate marker for the identification of cccDNA transcription.

Most HBV-infected patients who attend our outpatient clinics are HBV e antigen (HBeAg) negative. HBeAg detection is essential to define the infection status and disease management (7). HBeAg seroconversion during treatment might be indicative of a low viral replicative phase (7), however, a direct marker of viral transcription is essential to prevent viral rebound after therapy interruption. The level of HBV core-related antigen (HBcrAg) has been considered a potential biomarker for early prediction of HBeAg seroconversion (8). HBcrAg is considered a surrogate marker for cccDNA transcriptional activity (9, 10). It consists of proteins produced by the core gene: core antigen, free or antibody-bound HBeAg, and the p22 pre-core protein. HBcrAg is a candidate marker for the classification of chronic infection together with low HBV DNA (<2000 IU/mL) (11), and persistent high HBcrAg titers are associated with HCC (12). However, HBcrAg detection tests are somewhat insensitive, and HBcrAg levels can be influenced by protein secretion from infected cells in addition to the size of the cccDNA reservoir, thus limiting its clinical utility. HBV RNA could be an optimal surrogate of cccDNA activity, being exclusively produced by the cccDNA minichromosome (13). HBV RNA is a natural product of the HBV replication cycle (14) and may be found in viral particles in serum as a result of variation in the efficiency of encapsulation and/or reverse transcription (5, 15). HBV RNA levels in the blood vary during chronic hepatitis and depend on the clinical disease stage (16, 17). This new marker has been proposed as an early predictor of HBeAg seroconversion (18) and as a factor in the classification of HBeAg chronic infection (17). Historically, HBV RNA in blood has been quantified using different in-house real-time reverse-transcription PCR RT-PCR techniques, with a sensitivity of approximately 1.6 log_10_ U/mL or 2 log_10_ copies/mL (19–21), and suboptimal interlaboratory reproducibility.

Here, we present results from a study of naive and NA-treated HBV infected patients using a reproducible, highly sensitive, and fully automated method to quantify circulating HBV RNA (limit of detection [LOD]=5 cps/mL and lower limit of quantification [LLOQ]=10 cps/mL). We evaluated the prognostic value of this marker for reaching a low HBsAg titer (≤100 IU/mL) during anti-HBV therapy.

## RESULTS

### Viral markers in naive patients.

**FIG 1:**
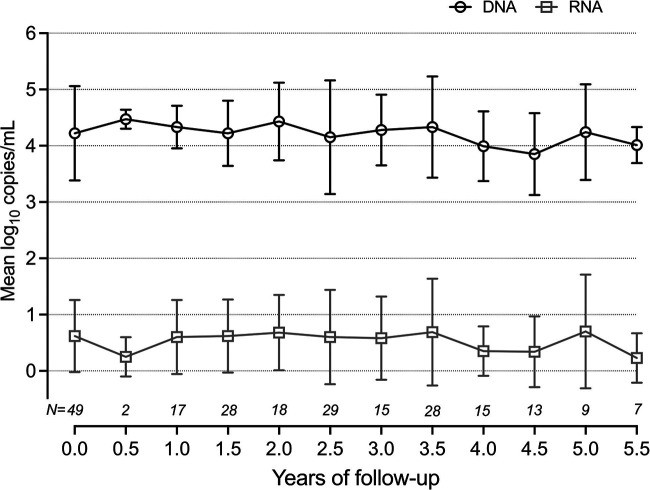
HBV DNA and HBV RNA trends over time in naive patients. HBV DNA (black open circles) and HBV RNA (gray open squares) titers (log_10_ copies/mL) throughout follow-up in treatment-naive patients. The number of patients (N) at each time point is shown in italics above the x-axis.

Plasma samples with valid (no errors during quantification) RNA results (*n* = 232) were collected from 50 antiviral drug-naive patients during the study period. HBV RNA was detectable (>LOD) in 120 samples from 43 patients. Mean HBV DNA and HBV RNA loads remained essentially unchanged during the follow-up period (Fig. 1). DNA levels were substantially higher than RNA. The mean difference between the HBV DNA and RNA was 3.6 log_10_ copies/mL (*P* < 0.000; around 3 log_10_ if we considered HBV DNA in IU/mL). The two markers partially correlated with each other (rho = 0.7, *P* < 0.0001). A similar correlation was observed between HBcrAg and HBV RNA (rho = 0.5; *P* < 0.0001), whereas no correlation was observed between HBV RNA and qHBsAg (rho = 0.02; *P* = 0.72).

### Viral markers in treated patients.

Valid results for both DNA and RNA were obtained from a total of 191 samples from 52 patients who received NA treatment for up to 9 years, including baseline samples from 26 patients. Of the 165 on-treatment samples, DNA and RNA were both undetectable in 86, both detectable (including <LLOQ) in 19, RNA detected but DNA undetected in 27, and RNA undetected but DNA detected in 33. RNA titers partially correlated with HBV DNA (rho = 0.5; *P* < 0.0001) and to some degree with HBcrAg and qHBsAg (rho < 0.4; *P <* 0.001).

**FIG 2:**
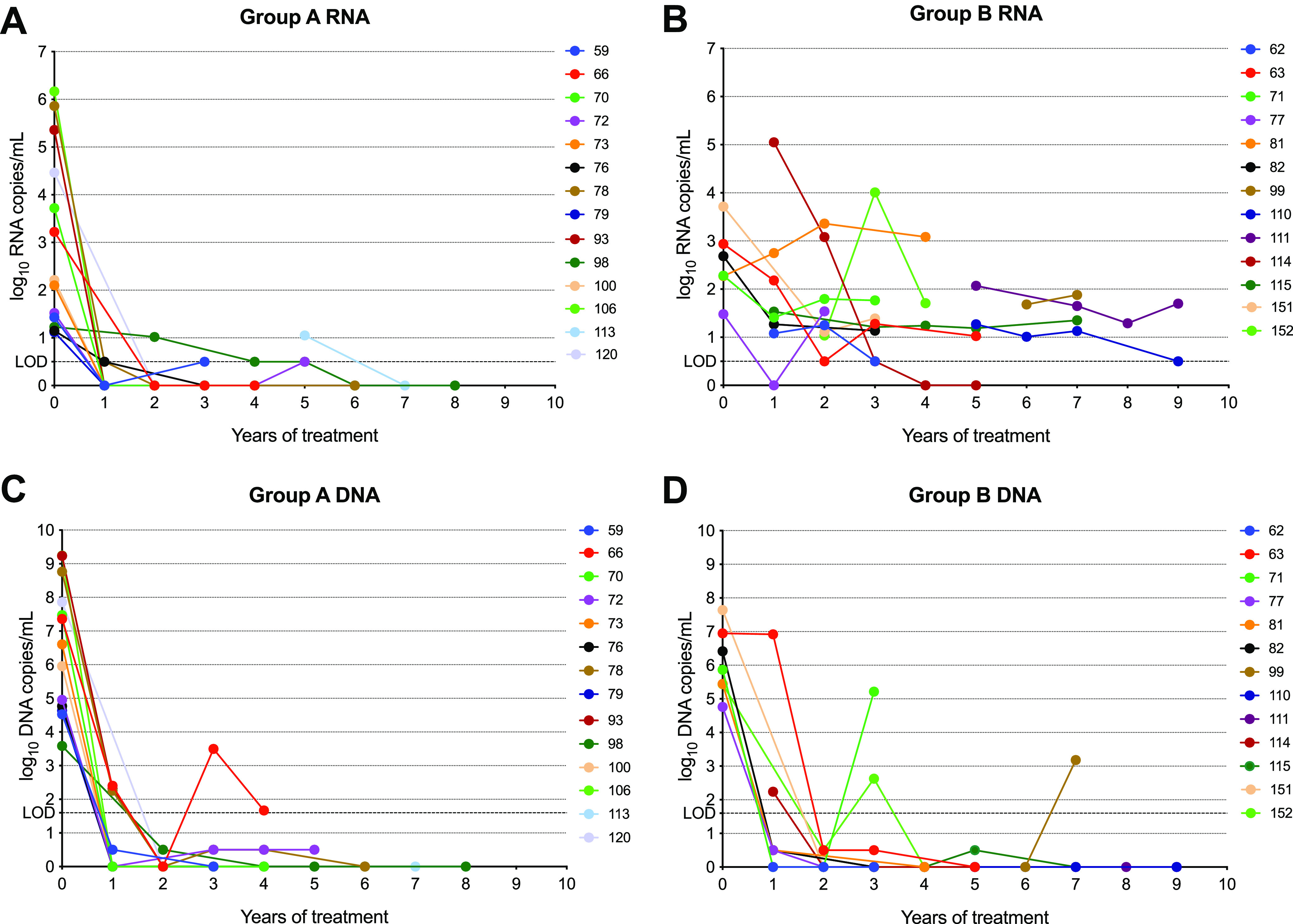
HBV RNA and HBV DNA during follow-up in NA-treated patients. The graphs show HBV RNA and DNA titers (log_10_ copies/mL) during years of treatment. Patients in group A ([A and C] for viral RNA and DNA, respectively) had HBV RNA in on-treatment samples below the lower limit of quantification (LLOQ), except for isolated blips in three patients. Patients in group B ([B and D] for viral RNA and DNA, respectively) had HBV RNA levels > LLOQ except for an isolated detectable but < LLOQ result for one patient. Patients with RNA < LLOQ at baseline and all on-treatment time points (*n* = 20) are not shown.

At baseline (before treatment), the difference between HBV DNA and HBV RNA was like that observed in naive patients (mean of differences: 3.6 log_10_ copies/mL; 2.9 if considering HBV DNA in IU/mL). Patients were grouped based on the patterns of HBV RNA during treatment, relative to baseline or the earliest sample with detectable RNA if the baseline sample was not available. In group A (*n* = 14), on-treatment RNA levels decreased and dropped below the LLOQ (Fig. 2A; patient 113, with an isolated blip when RNA was 1.05 log_10_ copies/mL, were retained in group A). In group B (*n* = 13), on-treatment RNA levels remained above the LLOQ (Fig. 2B). HBV DNA titers decreased rapidly with time on treatment in all excluding four patients that reached levels below the LLOQ after 2 to 3 years (Fig. 2C and D).

### HBsAg levels in HBV RNA and HBcrAg undetectable samples.

**FIG 3:**
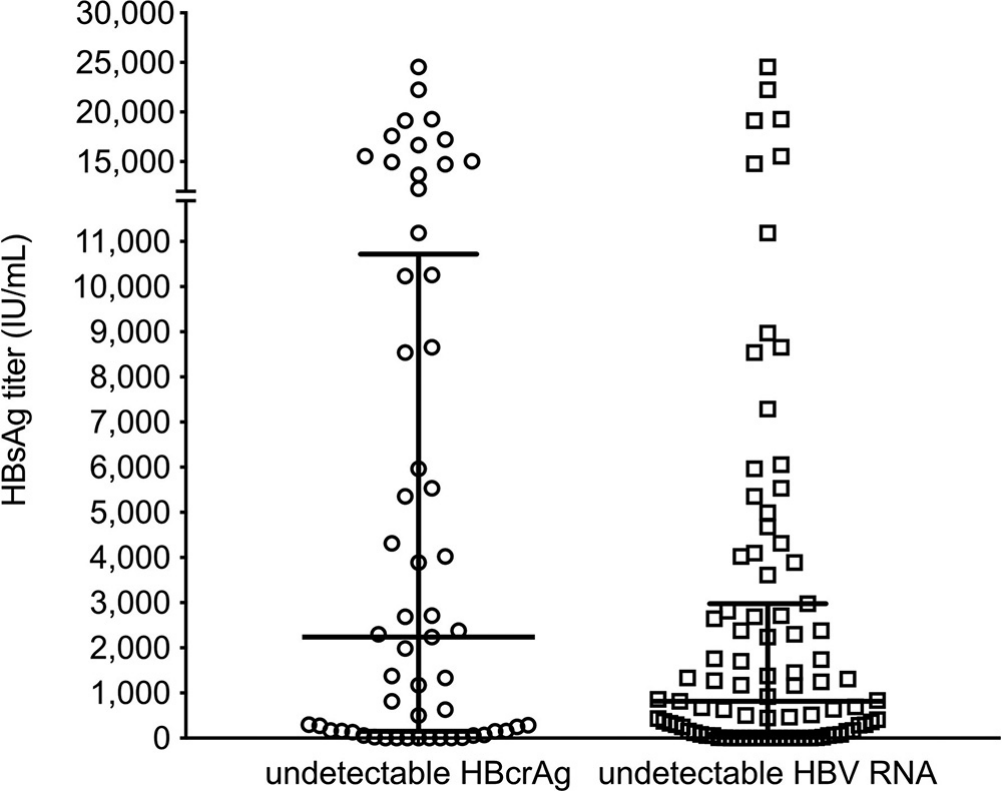
HBsAg titer in patients with undetectable HBcrAg or HBV RNA. The plot shows the HBsAg titer in those treated patients with undetectable HBcrAg (≤2.5 log_10_ U/mL) or undetectable HBV RNA (<LOD). Only those samples with both markers analyzed were included. Horizontal bars represent the median and interquartile range.

Considering that HBV RNA and HBcrAg are promising predictors of viral suppression and HBsAg loss in treated patients, we analyzed HBsAg levels in samples from treated patients with undetectable HBV RNA (<LOD) or HBcrAg (≤2.5 log_10_ U/mL; Fig. 3). Only those samples with both HBV RNA and HBcrAg analyzed were included in the analysis. The median HBsAg concentration in samples with undetectable HBcrAg (2237 IU/mL; *n* = 57) was 2.7-fold higher than in samples with undetectable HBV RNA (817 IU/mL, *n* = 91; Kruskal Wallis adjusted *P* = 0.012).

### HBV RNA as a predictor of HBsAg decrease.

To identify predictors of HBsAg decrease to low levels in treated patients, different parameters were analyzed by multivariate analysis. Only 12 of the treated patients reached a very low HBsAg titer (<100 IU/mL) at the end of the study. Different independent variables were considered, both categorical (sustained, detectable HBV RNA during follow-up, detectable HBV RNA at the time of earliest HBV DNA undetectability, undetectable HBcrAg at the end of follow-up, both undetectable HBV RNA and HBcrAg at the end of follow-up, previous antiviral treatment) and continuous (HBV RNA and years of treatment). Detectable HBV RNA was a negative predictor of HBsAg decrease to ≤100 IU/mL (*P* = 0.03; Table 1). As expected, the year of treatment was a positive predictor of HBsAg decrease (*P* = 0.03). Of note, the presence of constant HBV RNA levels during follow-up showed a tendency of association with the outcome although not statistically significant. Notably, HBcrAg undetectability (≤2.5 log_10_ U/mL) at the end of follow-up was not associated with the outcome.

**TABLE 1:** Predictors of HBsAg decrease in treated patients^*a*^

Categorical variables	Univariate *P* value	Multivariate *P* value	Odds ratio
Sustained, detectable HBV RNA	0.09		
Detectable HBV RNA	0.04	0.03	0.09
Undetectable HBcrAg	1		
Undetectable HBV RNA and HBcrAg	0.5		
Previous treatment	1		
Continuous variables			
HBV RNA	0.28		
Yrs of treatment	0.05	0.04	1.4

a *P* values were obtained by applying the Fisher test for categorical and binomial regression for continuous variables related to the outcome (HBsAg <1000 IU/mL). Only those variables with a *P* <0.05 were included in the multivariate logistic regression. *P* values were corrected by Wald correction. HBcrAg: HBV core-related.

## DISCUSSION

Circulating HBV RNA is a promising biomarker to study infection state and progression of HBV infection, being predominantly composed by pgRNA (22) which is a direct transcription product from cccDNA. Several different in-house procedures for HBV RNA quantification have been developed (23), however, they are usually characterized by a low sensitivity that restricts the study to those patients with HBV RNA titers higher than 100 copies/mL. Here, we used a novel, fully automated, and highly reproducible real-time HBV RNA RT-PCR quantification method with a low limit of quantification (10 copies/mL), specifically designed for a widely implemented analytical system (Cobas 6800/8800). The use of the Cobas 6800 system allows for the testing of multiple analytes in the same sample on one platform. Assays with increased sensitivity (lower LLOQ) enhance the ability to identify patients with detectable HBV RNA, which could be underestimated using other methods. As previously reported, the circulating HBV RNA measured with this system correlated with intracellular 3.5 kb HBV RNA and with the 3.5 kb RNA/cccDNA ratio, which is an indicator of cccDNA transcriptional activity (24). HBV RNA might be a useful marker to evaluate cccDNA activity in NA-treated patients when the measurement of DNA levels is unreliable.

In the treatment-naive patients studied here, DNA and RNA levels correlated with each other and were stable over time, maintaining a difference of ∼3.6 log_10_ copies/mL (∼4000-fold). Notably, HBV RNA and DNA assays have important differences, including amplified target, internal controls, and other procedural details, such that results may not be directly comparable. Of note, the main goal of this study was to describe trends over time in the two markers, and we observed that HBV RNA titer was lower than what has been previously observed. Previous studies have reported HBV DNA titers between one to 1000-fold higher than RNA (16, 19, 24). There are several possible explanations for the difference between our results and previous studies (25). There is some uncertainty about the relationship between DNA measured in IU/mL and copies/mL. If we measured DNA levels in IU/mL instead of copies/mL, the mean difference was 2.8 log_10_. The proportion of arbitrarily assigned values for results that were < LLOQ could also play a role. For methods with higher LOD and LLOQ, the arbitrary values assigned to these samples may be higher. Additionally, different RNA assays have different target locations and may be calibrated differently.

After the first year of treatment, HBV DNA (reflecting viremia and ongoing replication) declined dramatically, whereas, in some cases, the HBV RNA decrease was smaller. This is in accordance with previous studies, where higher circulating HBV RNA concentrations (20) and delayed reduction (15) were observed. Notably, some successfully treated patients had detectable and sustained HBV RNA levels for years, even when HBV DNA is undetectable. The presence of HBV RNA after prolonged HBV DNA suppression highlights the potential role of interference with cccDNA expression using the new therapeutic approaches based on gene silencing (26).

A relevant percentage of successfully treated patients with viral suppression on therapy experience viral relapses after treatment is stopped (27, 28). To better manage therapy discontinuation, prevent viral relapses, and achieve a functional cure, optimal viral markers of cccDNA activity are required.

HBcrAg and HBV RNA can be detected even after years of HBV DNA suppression (29) and undetectable HBcrAg and HBV RNA are negative predictors of viral relapses in NA-treated patients (18, 20, 29). An end-of-treatment HBsAg level <100 IU/mL helped to identify patients with a greater likelihood of HBsAg clearance (30) and limited probability of viral relapse (21).

The HBsAg titer in samples with undetectable HBcrAg was around four times higher than in samples with undetectable HBV RNA. The detectable titer of HBV RNA at HBV DNA undetectability was negatively associated with HBsAg concentration <100 IU/mL, whereas no association was observed when considering HBcrAg alone or in combination with viral RNA undetectability at the end of treatment. Moreover, the presence of constant levels of HBV RNA even in the presence of viral suppression could be another negative factor associated with HBsAg decrease, although the relationship was not statistically significant. Patients with a positive response to interferon-based treatment were characterized by an early decline of HBV RNA during treatment (31–33). Very low HBV RNA levels and undetectable HBV DNA could be a predictor of a durable antiviral response after NA treatment discontinuation (34). Patients with HBsAg titer ≤1000 IU/mL and undetectable HBV RNA at the end of treatment had a lower rate of viral relapse after 2 years off-therapy (35). As previously reported by Carey et al. (29), HBV RNA and HBcrAg are sensitive biomarkers of continued transcription from cccDNA in HBeAg negative patients, despite marked HBV DNA suppression by NAs. In the same study, patients that experienced severe ALT flares after treatment withdrawal had higher HBV RNA or HBcrAg when therapy was stopped (29).

These conclusions are subject to some limitations. Due to sample volume constraints, it was not possible to analyze HBcrAg in all samples in our study, and the number of patients that presented with constant HBV RNA levels was low. This study was focused on HBeAg-negative patients, which represent most HBV-infected patients in Spain and the entire Mediterranean basin. Furthermore, this was a real-life study in which the number of newly treated patients included was limited by the availability of appropriate baseline samples. Further studies with a larger cohort that includes more HBeAg-positive, naive, and treated patients, as well as patients undergoing treatment interruption, are required to evaluate the utility of HBV RNA quantification in treated patients as a predictor of viral relapse.

In summary, we used a new fully automated method for quantification of circulating HBV RNA that enables its detection with high sensitivity that could be applied in the general management of HBV infection. We observed that the difference between HBV DNA and HBV RNA in naive patients was greater than previously reported. In some NA-treated patients, HBV RNA was still detectable after years of successful treatment, suggesting persistent viral transcription from cccDNA. Of note, HBV RNA undetectability at the time of HBV DNA suppression could be related to a lower HBsAg level during follow-up, suggesting a reduction of transcription that could help identify those treated patients that may achieve a sustained viral suppression.

## MATERIALS AND METHODS

### Viral marker quantification.

HBsAg and HBeAg were analyzed by chemiluminescence immunoassay by Cobas 8000 (Roche Molecular Systems, Pleasanton, CA). HBcrAg was quantified by Lumipulse G chemiluminescence assay (Fujirebio). HBcrAg titers ≤ 2.5 log_10_ U/mL were considered undetectable.

HBV DNA was measured with the Cobas HBV test on the Cobas 6800 automated system (Roche). The lower limit of quantitation (LLOQ) of this test is 10 IU/mL, and the LOD for plasma samples is 6.6 IU/mL (21). Of note, this assay enables viral DNA quantification without any overestimation due to the presence of circulating HBV RNA because it lacks a retro-transcription step (36). Samples with detectable DNA results below the LLOQ were assigned an arbitrary value of 0.5 log_10_ IU/mL. Those with results below the LOD were assigned an arbitrary value of 0 log_10_ IU/mL.

Circulating HBV RNA levels were measured with the Cobas HBV RNA real-time quantitative RT-PCR assay for use on the Cobas 6800/8800 Systems (Roche) Molecular Systems (36, 37). This assay has an LLOQ of 10 copies/mL and a linear range of 10 to 10^9^ copies/mL on armored RNA (37). HBV RNA was considered undetectable when the result was less than 0.5 log_10_ copies/mL. Samples with detectable RNA results below the LLOQ were assigned an arbitrary value of 0.5 log_10_ copies/mL. Those with undetectable results were assigned an arbitrary value of 0 log_10_ copies/mL.

Comparison of HBV RNA and DNA levels is complicated by the lack of an international standard to define IU for RNA assays. Moreover, both assays are different in terms of target, internal control, and sample volume, which further limits the comparison between the markers. However, this study mainly aimed to analyze the HBV RNA trend over time and its association with HBV DNA. To facilitate this purpose, we converted DNA test results from IU/mL to copies/mL using the conversion factor of 5.82 (38).

### Patient characteristics.

Patients attending the outpatient clinics of Vall d’Hebron Hospital (Barcelona, Spain) were informed about the aims of the project and signed an informed consent form. Plasma samples were collected at various times during follow-up and analyzed for HIV, hepatitis C, and delta virus coinfection. HBV-monoinfected patients were included in the anonymized data set (see Tables S1 and S2 for additional details).

A total of 102 HBV DNA and HBsAg positive patients (both markers ≥ assay LOD) were included in the study: 50 were untreated, and 52 were treated with NA antiviral therapy. All the naive patients were HBeAg-negative, whereas five of 52 treated patients were HBeAg-positive at baseline, but rapidly became HBeAg-negative during the first year of treatment. The duration of follow-up ranged from zero to 5.5 years for untreated patients, and zero to 9 years for treated patients. Patient samples were grouped according to time since the first sample (untreated) or treatment initiation (treated) by rounding up to the nearest bi-annual (untreated) or annual (treated) time point. Among the treated patients, 45 patients initiated treatment with tenofovir disproxil fumarate (TDF), three with entecavir, one received a combination of entecavir and TDF, one TDF and emtricitabine, and for two the nucleos(t)ide analog (NA) used was not specified. Twenty-five of the treated patients were already on treatment at the time of study enrollment. HBV RNA could not be quantified in pretreatment samples from these patients because the previously collected samples had not been stored under conditions required for the preservation of HBV RNA. Two of the 52 treated patients lost detectable HBsAg during follow-up.

### Statistical analysis.

Means and medians were compared using a Mann-Whitney or Kruskal-Wallis test, respectively. *P* values were adjusted by applying the Bonferroni correction for multiple comparisons. Correlations were analyzed by the Spearman test. Multivariate analysis to detect possible predictive factors of HBsAg decrease to below 100 IU/mL was performed based on patterns of DNA and RNA detectability. Patients were categorized as “RNA detectable” if the RNA result was greater than the LOD at the earliest time point after treatment with undetectable HBV DNA, or as “sustained, detectable HBV RNA” if HBV RNA decreased by <0.5 log_10_ starting from the earliest time point after treatment with undetectable HBV DNA. Categorical variables were studied using the Fisher Exact test, whereas continuous variables were analyzed by applying a binomial regression. Only those variables with a *P <* 0.05 were included in the multivariate logistic regression analysis. All statistical analysis was carried out with R software (version 3.3.3; https://www.r-project.org/).

## References

[1] World Health Organization. 2021. Hepatitis B. https://www.who.int/news-room/fact-sheets/detail/hepatitis-b. Accessed September 23, 2021.

[2] Nguyen MH, Wong G, Gane E, Kao JH, Dusheiko G. 2020. Hepatitis B virus: advances in prevention, diagnosis, and therapy. Clin Microbiol Rev 33:1–38. doi:10.1128/CMR.00046-19. PMC7048015 32102898

[3] Tu T, Zhang H, Urban S, Zoulim F, Grandgenett DP. 2021. Hepatitis B virus DNA integration: in vitro models for investigating viral pathogenesis and persistence. Viruses 13:180. doi:10.3390/v13020180. 33530322 PMC7911709

[4] Tu T, Budzinska MA, Vondran FWR, Shackel NA, Urban S. 2018. Hepatitis B virus DNA integration occurs early in the viral life cycle in an *in vitro* infection model via NTCP-dependent uptake of enveloped virus particles. J Virol 92:e02007-17. doi:10.1128/JVI.02007-17. 29437961 PMC5952132

[5] Hu J, Liu K. 2017. Complete and incomplete hepatitis B virus particles: formation, function, and application. Viruses 9:56. doi:10.3390/v9030056. 28335554 PMC5371811

[6] Kha-Tu Ho J, Jeevan-Raj B, Netter H-J. 2020. Hepatitis B virus (HBV) subviral particles as protective vaccines and vaccine platforms. Viruses 12:126–126. doi:10.3390/v12020126. 31973017 PMC7077199

[7] European Association for the Study of the Liver. 2017. EASL 2017 clinical practice guidelines on the management of hepatitis b virus infection. J Hepatology 67:370–398. doi:10.1016/j.jhep.2017.03.021. 28427875

[8] Coffin CS, Zhou K, Terrault NA. 2019. New and old biomarkers for diagnosis and management of chronic hepatitis B virus infection. Gastroenterology 156:355–368.e3. doi:10.1053/j.gastro.2018.11.037. 30472225 PMC6433165

[9] Testoni B, Lebossé F, Scholtes C, Berby F, Miaglia C, Subic M, Loglio A, Facchetti F, Lampertico P, Levrero M, Zoulim F. 2019. Serum hepatitis B core-related antigen (HBcrAg) correlates with covalently closed circular DNA transcriptional activity in chronic hepatitis B patients. J Hepatology 70:615–625. doi:10.1016/j.jhep.2018.11.030. 30529504

[10] Inoue T, Tanaka Y. 2019. The role of hepatitis B core-related antigen. Genes 10:357. doi:10.3390/genes10050357. 31075974 PMC6562807

[11] Riveiro-Barciela M, Bes M, Rodríguez-Frías F, Tabernero D, Ruiz A, Casillas R, Vidal-González J, Homs M, Nieto L, Sauleda S, Esteban R, Buti M. 2017. Serum hepatitis B core-related antigen is more accurate than hepatitis B surface antigen to identify inactive carriers, regardless of hepatitis B virus genotype. Clin Microbiol Infect 23:860–867. doi:10.1016/j.cmi.2017.03.003. 28288829

[12] Tada T, Kumada T, Toyoda H, Kiriyama S, Tanikawa M, Hisanaga Y, Kanamori A, Kitabatake S, Yama T, Tanaka J. 2016. HBcrAg predicts hepatocellular carcinoma development: an analysis using time-dependent receiver operating characteristics. J Hepatol 65:48–56. doi:10.1016/j.jhep.2016.03.013. 27034253

[13] Giersch K, Allweiss L, Volz T, Dandri M, Lütgehetmann M. 2017. Serum HBV pgRNA as a clinical marker for cccDNA activity. J Hepatol 66:460–462. doi:10.1016/j.jhep.2016.09.028. 27826059

[14] Prakash K, Rydell GE, Larsson SB, Andersson M, Norkrans G, Norder H, Lindh M. 2018. High serum levels of pregenomic RNA reflect frequently failing reverse transcription in hepatitis B virus particles. Virol J 15:86. doi:10.1186/s12985-018-0994-7. 29764511 PMC5952638

[15] Wang J, Yu Y, Li G, Shen C, Meng Z, Zheng J, Jia Y, Chen S, Zhang X, Zhu M, Zheng J, Song Z, Wu J, Shao L, Qian P, Mao X, Wang X, Huang Y, Zhao C, Zhang J, Qiu C, Zhang W. 2018. Relationship between serum HBV-RNA levels and intrahepatic viral as well as histologic activity markers in entecavir-treated patients. J Hepatology 68:16–24. doi:10.1016/j.jhep.2017.08.021. 28870671

[16] Wang J, Yu Y, Li G, Shen C, Li J, Chen S, Zhang X, Zhu M, Zheng J, Song Z, Wu J, Shao L, Meng Z, Wang X, Huang Y, Zhang J, Qiu C, Zhang W. 2018. Natural history of serum HBV-RNA in chronic HBV infection. J Viral Hepat 25:1038–1047. doi:10.1111/jvh.12908. 29633430

[17] Liu Y, Jiang M, Xue J, Yan H, Liang X. 2019. Serum HBV RNA quantification: useful for monitoring natural history of chronic hepatitis B infection. BMC Gastroenterol 19:53. doi:10.1186/s12876-019-0966-4. 30991954 PMC6469196

[18] van Bömmel F, Bartens A, Mysickova A, Hofmann J, Krüger DH, Berg T, Edelmann A. 2015. Serum hepatitis B virus RNA levels as an early predictor of hepatitis B envelope antigen seroconversion during treatment with polymerase inhibitors. Hepatology 61:66–76. doi:10.1002/hep.27381. 25132147

[19] Kaewdech A, Tangkijvanich P, Sripongpun P, Witeerungrot T, Jandee S, Tanaka Y, Piratvisuth T. 2020. Hepatitis B surface antigen, core-related antigen and HBV RNA: predicting clinical relapse after NA therapy discontinuation. Liver International 40:2961–2971. doi:10.1111/liv.14606. 32668074

[20] Butler EK, Gersch J, McNamara A, Luk K-C, Holzmayer V, de Medina M, Schiff E, Kuhns M, Cloherty GA. 2018. HBV serum DNA and RNA levels in nucleos(t)ide analogue-treated or untreated patients during chronic and acute infection. Hepatology 68:2106–2117. doi:10.1002/hep.30082. 29734472

[21] Seto W-K, Liu KS, Mak L-Y, Cloherty G, Wong DK-H, Gersch J, Lam Y-F, Cheung K-S, Chow N, Ko K-L, To W-P, Fung J, Yuen M-F. 2021. Role of serum HBV RNA and hepatitis B surface antigen levels in identifying Asian patients with chronic hepatitis B suitable for entecavir cessation. Gut 70:775–779. doi:10.1136/gutjnl-2020-321116. 32759300

[22] Anderson M, Gersch J, Luk KC, Dawson G, Carey I, Agarwal K, Shah P, Dusheiko G, Lau D, Cloherty G. 2021. Circulating pregenomic hepatitis B virus RNA is primarily full-length in chronic hepatitis B patients undergoing nucleos(t)ide analogue therapy. Clin Infect Dis 72:2029–2031. doi:10.1093/cid/ciaa1015. 32687164

[23] Liu S, Zhou B, Valdes JD, Sun J, Guo H. 2019. Serum hepatitis B virus RNA: a new potential biomarker for chronic hepatitis B virus infection. Hepatology 69:1816–1827. doi:10.1002/hep.30325. 30362148 PMC6438723

[24] Testoni B, Scholtes C, Plissonnier M-L, Berby F, Facchetti F, Villeret F, Loglio A, Scott B, Wang L, Hamilton A, Heil M, Lampertico P, Levrero M, Zoulim F. 2020. Circulating HBV RNA correlates with intrahepatic covalently closed circular DNA (cccDNA) levels and activity in untreated chronic hepatitis B (CHB) patients. J Hepatol 75:S713–S714.

[25] Tabernero D, Cortese MF, Rando-Segura A, Buti M, Rodríguez-Frías F. 2022. Letter to the Editor: standardization of HBV RNA assay for the different phases of chronic hepatitis B is essential. Hepatology 75:501–502. doi:10.1002/hep.32227. 34758113

[26] Bloom K, Maepa MB, Ely A, Arbuthnot P. 2018. Gene therapy for chronic HBV-can we eliminate cccDNA? Genes 9:207. doi:10.3390/genes9040207. 29649127 PMC5924549

[27] Papatheodoridis GV, Rigopoulou EI, Papatheodoridi M, Zachou K, Xourafas V, Gatselis N, Hadziyannis E, Vlachogiannakos J, Manolakopoulos S, Dalekos GN. 2018. Daring-B: discontinuation of effective entecavir or tenofovir disoproxil fumarate long-term therapy before HBsAg loss in non-cirrhotic HBeAg-negative chronic hepatitis B. Antivir Ther 23:677–685. doi:10.3851/IMP3256. 30044765

[28] Ma TL, Hu TH, Hung CH, Wang JH, Lu SN, Chen CH. 2019. Incidence and predictors of retreatment in chronic hepatitis B patients after discontinuation of entecavir or tenofovir treatment. PLoS One 14:e0222221. doi:10.1371/journal.pone.0222221. 31584951 PMC6777800

[29] Carey I, Gersch J, Wang B, Moigboi C, Kuhns M, Cloherty G, Dusheiko G, Agarwal K. 2020. Pregenomic HBV RNA and hepatitis B core-related antigen predict outcomes in hepatitis B e antigen-negative chronic hepatitis B patients suppressed on nucleos(t)ide analogue therapy. Hepatology 72:42–57. doi:10.1002/hep.31026. 31701544

[30] Kuo MT, Tseng PL, Chou YP, Chang KC, Tsai MC, Kuo YH, Hu TH, Hung CH, Wang JH, Lu SN, Cheng CH. 2018. Role of hepatitis B surface antigen in hepatitis B virus relapse after entecavir or tenofovir prophylaxis in patients undergoing cancer chemotherapy. J Gastroenterology and Hepatology 33:1766–1772. doi:10.1111/jgh.14142. 29514418

[31] Farag MS, van Campenhout MJH, Pfefferkorn M, Fischer J, Deichsel D, Boonstra A, van Vuuren AJ, Ferenci P, Feld JJ, Berg T, Hansen BE, van Bömmel F, Janssen HLA. 2021. Hepatitis B virus RNA as Early Predictor for Response to PEGylated Interferon Alfa in HBeAg Negative Chronic Hepatitis B. Clinical Infectious Diseases 72:202–211. doi:10.1093/cid/ciaa013. 31912157

[32] Limothai U, Chuaypen N, Poovorawan K, Chotiyaputta W, Tanwandee T, Poovorawan Y, Tangkijvanich P. 2019. Baseline and kinetics of serum hepatitis B virus RNA predict response to pegylated interferon-based therapy in patients with hepatitis B e antigen-negative chronic hepatitis B. J Viral Hepat 26:1481–1488. doi:10.1111/jvh.13195. 31446638

[33] van Campenhout MJH, van Bömmel F, Pfefferkorn M, Fischer J, Deichsel D, Boonstra A, van Vuuren AJ, Berg T, Hansen BE, Janssen HLA. 2020. Serum hepatitis B virus RNA predicts response to peginterferon treatment in HBeAg-positive chronic hepatitis B. Randomized Controlled Trial 27:610–619. doi:10.1111/jvh.13272. PMC7383601 32052503

[34] Rong F, Bin Z, Min X, Deming T, Junqi N, Hao W, Hong R, Xinyue C, Maorong W, Qin N, Guangfeng S, Jifang S, Hong T, Xuefan B, Shi L, Fengmin L, Jie P, Jian S, Qing X, Jinlin H, Mobin W, Shijun C, Yanyan Y, Hong M, Jun C, Hongfei Z, Huimin L, Zhiliang G, Xiaoguang D. 2020. Association between negative results from tests for HBV DNA and RNA and durability of response after discontinuation of nucles(t)ide analogue therapy. Clinical Gastroenterology and Hepatology 18:719–727.e7. doi:10.1016/j.cgh.2019.07.046. 31362119

[35] Liu Y, Xue J, Liao W, Yan H, Liang X. 2020. Serum HBV RNA dynamic and drug withdrawal predictor value in patients with chronic HBV infection on long-term nucleos(t)ide Analogue (NA) Therapy. J Clin Gastroenterol 54:E73–E82. doi:10.1097/MCG.0000000000001376. 32604147 PMC7458089

[36] Maasoumy B, Geretti AM, Frontzek A, Austin H, Aretzweiler G, Garcia-Álvarez M, Leuchter S, Simon CO, Marins EG, Canchola JA, Cornberg M, Delgado R, Wedemeyer H. 2020. HBV-RNA Co-amplification may influence HBV DNA viral load determination. Hepatol Commun 4:983–997. doi:10.1002/hep4.1520. 32626831 PMC7327219

[37] Scholtès C, Hamilton A, Scott B, Wang L, Plissonnier M, Berby F, French J, Charre C, Testoni B, Blair A, Paturel A, Subic M, Hoppler M, Lankenau A, Grubenmann A, Levrero M, Heil M, Zoulim F. 2020. Performance of a novel automated assay for the detection and quantification of HBV pregenomic RNA/circulating RNAs in chronic HBV patients. Hepatology 72:447.

[38] Palom A, Sopena S, Riveiro‐Barciela M, Carvalho‐Gomes A, Madejón A, Rodriguez‐Tajes S, Roade L, García‐Eliz M, García‐Samaniego J, Lens S, Berenguer‐Hayme M. 2021. One-quarter of chronic hepatitis D patients reach HDV-RNA decline or undetectability during the natural course of the disease. Alimentary Pharmacology & Therapeutics 54:462–469. doi:10.1111/apt.16485. 34181772

