# Supplementary material for: Standardized Hepatitis B Virus RNA Quantification in Untreated and Treated Chronic Patients: a Promising Marker of Infection Follow-Up
Source: Microbiol Spectr. 2022 Apr 4;10(2):e02149-21. doi: 10.1128/spectrum.02149-21 (PMC9045303; doi:10.1128/spectrum.02149-21)
Supplement: SUPPLEMENTAL FILE 1 — Supplemental material. Download SPECTRUM02149-21_Supp_1_seq3.pdf, PDF file, 0.1 MB [file spectrum02149-21_supp_1_seq3.pdf]

Supplementary material for

Standardized HBV RNA quantification in untreated and treated chronic patients: a promising marker of infection follow-up.

Cortese et al.

Table S1. Clinical characteristics of naïve patients.

Follow-up (semester)	N	qHBsAg mean ± SD (log10 IU/mL)	qHBsAg Undetect-able* (n)	HBcrAg Mean ± SD (log10 U/mL)	HBcrAg Undetect-able* (n)	HBcrAg <LLOQ (n)	HBcrAg No result (n)	FIB-4 (mean ± SD)	ALT (U/L) (mean ± SD)	AST (U/L) (mean ± SD)
Baseline	60	4.0 ± 4.1	0	2.4 ± 0.6	38	14	3	1.2 ± 0.4	29.7 ± 15.2	29.8 ± 9.5
1	23	3.9 ± 4.0	1	3.0 ± 1.0	2	1	17	1.2 ± 0.4	26.5 ± 11.4	28.1 ± 10.2
2	33	3.7 ± 3.8	0	2.3 ± 0.4	5	3	25	1.1 ± 0.3	24.4 ± 11.7	25.6 ± 6.5
3	22	4.2 ± 4.3	0	2.3 ± 0.4	9	1	11	1.0 ± 0.5	29.9 ± 17.2	29.1 ± 10.0
4	29	3.6 ± 3.7	0	2.5 ± 0.5	6	3	19	1.1 ± 0.4	27.3 ± 11.4	27.3 ± 7.1
5	15	4.1 ± 4.3	0	2.3 ± 0.5	3	1	10	1.2 ± 0.5	25.3 ± 9.2	26.5 ± 6.4
6	22	3.9 ± 4.2	0	2.4 ± 0.9	10	1	10	1.2 ± 0.4	26.9 ± 14.0	27.1 ± 9.3
7	16	3.8 ± 4.0	1	2.2 ± 0.4	8	1	6	1.3 ± 0.4	28.1 ± 14.9	27.8 ± 6.2
8	15	3.8 ± 3.8	0	2.3 ± 0.5	9	0	5	1.2 ± 0.4	26.1 ± 7.0	26.1 ± 5.0
9	5	3.8 ± 3.8	0	2.0 ± 0.0	1	0	4	1.2 ± 0.4	23.2 ± 17.3	23.4 ± 8.1
>10	9	3.3 ± 2.9	0	2.0 ± 0.0	7	0	0	1.6 ± 0.3	18.0 ± 6.1	22.2 ± 3.1

*Samples with a qHBsAg and HBcrAg below 0.05 U/mL and 2.5 $\log_{10}$ U/mL respectively were considered undetectable.

ALT: alanine aminotransferase; AST: aspartate aminotransferase; FIB-4: Fibrosis-4 index; HBcrAg: HBV core-related Antigen; LLOQ: Lower limit of quantification; qHBsAg: quantitative HBV surface Antigen; SD: standard deviation.

Table S2. Clinical characteristics of treated patients.

		qHBsAg		HBcrAg						
**Follow-up time (years)**	**N**	**mean ± SD ($\log_{10}$ IU/mL)**	**Undetect-able* (n)**	**mean ± SD ($\log_{10}$ U/mL)**	**Undetect-able* (n)**	**<LLOQ (n)**	**No result (n)**	**FIB-4 (mean ± SD)**	**ALT (U/L) (mean ± SD)**	**AST (U/L) (mean ± SD)**
Baseline	21	4.0 ± 4.0	0	3.4 ± 1.4	6	3	4	1.3 ± 0.6	43.9 ± 32.1	35.1 ± 18.2
1	24	4.0 ± 3.6	0	2.6 ± 0.8	10	4	4	1.5 ± 1.0	32.8 ± 17.1	30.3 ± 11.8
2	22	3.6 ± 4.0	0	3.0 ± 1.0	8	3	4	1.8 ± 1.7	30.3 ± 10.3	32.9 ± 15.4
3	11	4.1 ± 3.2	1†	3.5 ± 0.9	1	1	4	1.4 ± 0.8	29.7 ± 9.3	29.6 ± 7.6
4	13	3.1 ± 3.0	1†	3.0 ± 0.9	2	2	6	2.3 ± 1.4	28.3 ± 10.2	30.5 ± 7.4
5	17	3.1 ± 3.5	0	2.7 ± 0.7	4	2	7	3.1 ± 2.2	28.6 ± 15	32.0 ± 12.3
6	8	3.4 ± 3.9	0	2.8 ± 1.3	4	1	3	2.5 ± 1.9	24.5 ± 10.9	25.6 ± 7.6
7	13	3.7 ± 3.8	0	2.9 ± 1.1	3	3	4	3.1 ± 1.8	28.1 ± 13.0	35.5 ± 16.8
8	9	3.5 ± 4.0	1	2.6 ± 0.5	3	4	1	2.9 ± 1.8	25.9 ± 12.0	29.9 ± 9.3
9	5	3.8 ± 3.9	0	2.1 ± 0.3	4	1	0	4.9 ± 3.3	34.0 ± 12.0	40.2 ± 13.2
>10	4	3.7 ± 3.7	0	2.4 ± 0.4	2	2	0	4.1 ± 3.4	24.5 ± 10.3	30.3 ± 14.1

*Samples with a qHBsAg and HBcrAg respectively below ≤0.05 U/mL and 2.5 log_{10} U/mL were considered undetectable.

†Results correspond to the same patient. Only longitudinal samples were included.
